# Data-driven clinical decision support tool for diagnosing mild cognitive impairment in Parkinson’s disease

**DOI:** 10.1038/s41531-025-01222-6

**Published:** 2026-01-12

**Authors:** Gabriel Martínez Tirado, Patricia Martins Conde, Stefano Sapienza, Holger Fröhlich, Claire Pauly, Valerie E. Schröder, Sonja Jónsdóttir, Olena Tsurkalenko, Rejko Krüger, Jochen Klucken

**Affiliations:** 1 Luxembourg Centre for Systems Biomedicine (LCSB), University of Luxembourg, Esch-sur-Alzette, Luxembourg; 2 Bonn-Aachen International Center for Information Technology (B-IT), Rheinische Friedrich-Wilhelms-Universität Bonn, Bonn, Germany; 3 Department of Bioinformatics, Fraunhofer Institute for Algorithms and Scientific Computing (SCAI), Sankt Augustin, Germany; 4 Centre Hospitalier de Luxembourg, Strassen, Luxembourg; 5 Luxembourg Institute of Health (LIH), Strassen, Luxembourg; 6 Laboratoire National de Santé, Dudelange, Luxembourg; 7 Faculty of Science, Technology and Medicine, University of Luxembourg, Esch-sur-Alzette, Luxembourg; 8 Luxembourg Center of Neuropathology, Dudelange, Luxembourg; 9 Department of Life Sciences and Medicine, University of Luxembourg, Esch-sur-Alzette, Luxembourg; 10 Department of Epidemiology, CAPHRI School for Public Health and Primary Care, Maastricht University Medical Centre+, Maastricht, the Netherlands; 11 Centre Hospitalier Emile Mayrisch, Esch-sur-Alzette, Luxembourg; 12 Parkinson Luxembourg Association, Leudelange, Luxembourg; 13 Association of Physiotherapists in Parkinsons Disease Europe, Esch-sur-Alzette, Luxembourg; 14 Private practice, Ettelbruck, Luxembourg; 15 Private practice, Luxembourg, Luxembourg

**Keywords:** Biomarkers, Diseases, Neurology, Neuroscience

## Abstract

Parkinson’s disease (PD) is a neurodegenerative condition that may affect both motor and cognitive function. Mild cognitive impairment (MCI) is a known risk factor for the progression to dementia in the later stages of the disease. Lengthy and time-consuming neuropsychological assessments, by trained experts, often make MCI diagnosis impractical in routine care. In this context, machine learning (ML) may offer promising support for MCI diagnosis. Thus, we analysed longitudinal data from 115 people with Parkinson’s disease (PwPD) and 226 healthy control participants from the Luxembourg Parkinson’s Study, combining ML with clinical data to support MCI diagnosis in PwPD. The data-driven model showed a non-inferior performance to the clinical diagnostic reference test (MDS PD-MCI Level II) and identified a subgroup of MCI individuals that was not captured by the clinical test. This finding suggests that ML models can complement clinical assessments, by facilitating the detection of MCI and complementing the diagnostic characterisation of PwPD.

## Introduction

Parkinson’s disease (PD) is the second most common neurodegenerative condition, affecting over 1% of the population aged 60 years and older^1^, with a worldwide prevalence that has been steadily increasing in recent decades^2^. PD is associated with both motor and non-motor symptoms, including cognitive impairment, depression, anxiety, and sleep disorders^3^. Among these, cognitive impairment is considered one of the most significant non-motor aspects of PD, as it significantly reduces patients’ quality of life by affecting attention, executive function, visuospatial abilities, and contributing to neuropsychiatric disturbances, ultimately leading to functional impairment^4–6^. This worsening in the cognitive functions also has a substantial impact on caregiver well-being.

Mild Cignitive Impairment (MCI) can be considered a transitional stage between normal cognition (NC) and dementia^7^. It is a clinically important syndrome due to both its high prevalence and its role as a significant risk factor for further cognitive decline. In PD, cognitive changes follow a similar continuum, often progressing from subjective cognitive decline (SCD) to Parkinson’s disease–specific MCI (PD-MCI) and, in some cases, to dementia. Therefore, MCI represents a clinically relevant condition for diagnosis and early detection of cognitive impairment in PD, enabling early intervention and monitoring of potential progression to dementia. As a result, the establishment of diagnostic criteria has long been a priority in the field.

Diagnostic definitions for MCI in the elderly have evolved over time. Early definitions of MCI focused primarily on memory impairment with preserved functional abilities^8^. However, current consensus highlights the need for in-depth neuropsychological evaluation spanning the full spectrum of cognitive domains, including attention, executive function, language, and visuospatial skills^9^.

A wide range of diagnostic tools exists for diagnosing MCI in PwPD, from brief screenings, testing global cognition to comprehensive neuropsychological assessments specifically tailored for PwPD^10^. Among brief assessments, the Montreal Cognitive Assessment (MoCA)^11^ and the Mini–Mental State Examination (MMSE)^12^ are the most widely used. The MoCA total score has shown greater sensitivity when compared to the MMSE for screening MCI and monitoring cognitive decline in PwPD^13,14^, although its limited granularity and suboptimal sensitivity remain a concern^15^. The MoCA total score, for instance, offers only a general overview of the cognitive function and lacks the sensitivity to detect subtle or domain-specific impairments^15^. This led to the development of clinical diagnostic criteria for MCI in PD by the “International Parkinson and Movement Disorder Society (MDS PD-MCI)” task force in 2012^16^. This criterion is divided into two levels of comprehensiveness: (a) level I (abbreviated assessment) and (b) level II (comprehensive assessment). The MDS PD-MCI Level II criteria require a comprehensive neuropsychological assessment of cognitive function across different domains to identify MCI and are widely recognised as the gold standard^17–21^. Although MDS PD-MCI Level II criteria are accepted in clinical research, its implementation into everyday healthcare procedures has certain drawbacks. It relies on cognitive assessment through neuropsychological tests, which are time-consuming, require expert interpretation, and are based on predefined rigid cut-off values^22,23^. Furthermore, there is currently no consensus on the optimal cutoff thresholds for identifying MCI in PwPD. Cutoff ranges from 1 to 2 standard deviations (SD) below the normative mean, are applied across one or multiple domains. This variation leads to significant differences in the proportion of PD-MCI cases identified in different studies^16,22^, often leading to diagnostic heterogeneity. This variability not only complicates clinical decision-making but also limits comparability across studies and settings. These limitations highlight the urgent need for alternative, more flexible approaches to improve MCI identification in PD.

Artificial Intelligence (AI) and its resulting machine learning (ML) applications are emerging as an essential tool in clinical diagnostics, particularly in assessing cognitive function and supporting the diagnosis of neurodegenerative diseases. By analysing and integrating clinical data from different sources, ML models have demonstrated the ability to detect subtle patterns in cognitive assessments, offering new approaches to improve early detection of cognitive impairment in neurodegenerative diseases^24,25^. This revolutionises traditional diagnostic approaches, making them more efficient, personalised, and scalable.

In Parkinson’s disease (PD), mild cognitive impairment (MCI) is an important stage for timely intervention, yet current diagnostic approaches can be time-consuming and may not fully capture patient variability. ML methods offer a way to complement neuropsychological evaluations by modelling complex relationships in clinical data that may relate to cognitive decline. Recent advances in ML, including ensemble learning methods and explainable AI, have shown potential to improve prediction accuracy and enhance interpretability, offering valuable insights for clinical decision-making^26–28^.

Building on these advancements and capabilities, this study aims to improve the diagnostic process of MCI by exploring the identification of MCI subgroups overlooked by the clinical diagnostic reference test. To this end, we generated a data-driven diagnostic model using cross-sectional retrospective clinical data from both healthy controls and PwPD with different levels of cognitive decline. Rather than replacing current clinical criteria, this approach aims to complement existing diagnostic practices and provide insights that could refine early detection strategies in PD.

## Results

### Descriptive statistics of the study data

A total of 115 PwPD and 226 healthy controls, from the Luxembourg Parkinson’s Study (LuxPark) cohort data, met the eligibility criteria for analysis. Both groups present differences in socio-demographic and clinical characteristics. Regarding socio-demographic characteristics, the PD group consisted of older individuals with a similar level of education compared to the healthy control group, and a higher proportion of males (71.55% vs 53.98%). Clinically, the PD group exhibited significantly greater impairments than healthy controls across the cognitive and motor function MDS-Unified Parkinson’s Disease Rating Scale (MDS-UPDRS III), activities of daily living (ADL), subjective cognitive complaints and neuropsychiatric symptoms (depression, apathy). When examining domain-specific cognitive performance, a heterogeneous pattern emerged: the PD group showed significantly worse performance in attention, executive function, and memory, whereas no significant differences were observed in language and visuospatial abilities (Table 1).

**Table 1:** Main clinical and socio-demographic characteristics of Parkinson’s disease (PD) and healthy control participants at the baseline visit in the LuxPark cohort

**Features**			**PD** **(*n*** **=** **115)** median (IQR), min-max or *n* (%)	**Healthy control** **(*n*** **=** **226)** median (IQR), min-max or *n* (%)	**Adjusted** ***p*-value**
Socio-demographic					
Age (years)			67.28 (15.10), 38–88	60.79 (14.50), 21–86	<0.001
Male			83 (72.17%)	122 (53.98%)	0.002
Education (years)			14 (5.5), 6–25	14.0 (6.0), 6–24	0.97
Clinical					
Global cognition					
MoCA total score			26.0 (4.0), 17–30	28.0 (3.0), 19–30	<0.001
Physician-rated cognitive impairment (MDS-UPDRS 1.1)		0 (Normal)	67 (58.6%)	166 (73.45%)	0.077
		1 (Slight Impairment)	39 (33.6%)	49 (21.68%)	
		2 (Mild impairment)	5 (4.3%)	8 (3.54%)	
		3 (Moderate Impairment)	2 (1.7%)	2 (0.89%)	
		4 (Severe impairment)	1 (0.86%)	0 (0%)	
Domain-specific neuropsychological assessments					
Attention	TMT-A (seconds)		44.0 (23.25), 19–113	35.0 (17.0), 12–150	<0.001
	Block span (Total score)		8.0 (3.0), 0–12	8.0 (2.0), 0–13	0.12
Executive	TMT(B-A) (seconds)		49.0 (16.0), 9–223	39.0 (28.25), 5–226	<0.001
	FAB		16.0 (3.0), 6–18	17.0 (2.0), 10–18	<0.001
Memory	Word list learning total score		22.0 (6.0), 5–29	23.0 (5.0), 11–30	0.002
	Word list delayed recall		7.0 (3.0), 0–10	8.0 (2.75), 0–10	<0.001
Visuospatial	Judgement of line orientation		25.0 (7.0), 5–30	26.0 (7.75), 0–30	0.12
	MoCA visuospatial		3.0 (1.0), 1–3	3.0 (0.0), 1–3	0.12
Language	Phonemic fluency S		9.0 (6.0), 1–21	10.0 (5.0), 2–21	0.093
	MoCA naming		3.0 (0.0), 1–3	3.0 (0.0), 2–3	0.37
Motor function					
Hoehn and Yahr			2.0 (0.0), 1–4	—	—
MDS-UPDRS III			28.0 (17.0), 3–74	2.0 (5.0), 0–20	<0.001
ADL					
PDQ-39 subitems 11–16			5.0 (7.0), 0–18	0.0 (0.0), 0–9	<0.001
Depressive symptoms					
Depression (BDI-I)			8.0 (6.0), 0–23	4.0 (1.0--7.25), 0–22	<0.001
Apathy (SAS)			12.0 (6.0), 1–29	9.0 (6.5–12.0), 0–25	<0.001
Subjective Cognitive Decline					
Subjective cognitive complaints (PDQ-39 subitems 30–33)			3.0 (4.0), 0–13	1.0 (0.0–2.75), 0–12	<0.001
Disease-related factors					
Disease duration (years)			2.0 (1.0–6.25), 0–22	—	—
Age at PD onset (years)			62.0 (16.25), 36–88	—	—

Clinical features include global cognition (e.g., MoCA total score), domain-specific neuropsychological performance (e.g., attention, executive functions, memory, visuospatial abilities, and language), PD staging (Hoehn and Yahr), motor function (e.g., MDS-UPDRS III), ADL and other disease-related factors such as disease duration and age at PD onset. Socio-demographic variables include age, sex, and years of education. Statistics are reported by median, interquartile range (IQR) and range (min–max); whereas sex, as a categorical feature, is reported by the percentage of male participants. The statistical significance of differences between the PD and healthy control groups was assessed using *p*-values, adjusted for multiple comparisons using the Benjamini-Hochberg method. *p*-. The alpha level was set at *α* = 0.05. Significant p-values are highlighted in bold. Distribution analyses were performed using the two-tailed *t*-test for numerical variables (normally distributed), two-tailed Mann–Whitney U test for numerical variables (not normally distributed) and Pearson’s chi-squared test for categorical variables. Abbreviations: *MoCA* Montreal Cognitive Assessment, *TMT* Trail Making Test, *FAB* Frontal Assessment Battery, *ADL* Activities of daily living, *PDQ-39* Parkinson's Disease questionnaire, *BDI-I* Beck Depression Inventory, *SAS* Starkstein Apathy Scale, *MDS-UPDRS* Movement Disorder Society-Unified Parkinson’s Disease Rating Scale.

### Data-driven model

To develop clinical decision support tools for diagnosing MCI, multiple data-driven models based on distinct clustering techniques (Gaussian Mixture Models (GMM), K-Means and Spectral Clustering (SC)) were built. An overview of the hyperparameters that were optimised and the best hyperparameter combination resulting from a grid search optimisation for each model is given in the Supplementary Table 1. Subsequently, the best models from the three algorithms were compared based on their prediction overlap with the clinical diagnostic reference test (MDS PD-MCI Level II). SC emerged as the most suitable method for diagnosing MCI, outperforming both methods, K-Means and GMM, in terms of sensitivity (recall) and area under the curve (AUC), while maintaining a comparable precision. A higher AUC indicates greater discriminatory power in distinguishing between NC and MCI, with SC achieving an AUC of 0.81, compared to 0.74 for GMM and 0.77 for K-Means. Additionally, the higher overall sensitivity of SC reflects its superior ability to correctly identify true positive MCI cases. More specifically, the sensitivity for MCI reached 0.97 — substantially higher than GMM (0.55) and K-Means (0.65). A comprehensive summary of the performance metrics is provided in Supplementary Table 2.

Having established SC as the most effective method for MCI diagnosis, we further examined the feature importance to elucidate which clinical and demographic factors most strongly influenced cluster separation for clinical interpretability, and to validate the clinical relevance of the identified subgroups. Domain-specific cognitive assessments were the primary drivers of cluster differentiation. Additionally, disease-related factors (e.g., age at PD diagnosis, disease duration), comorbidities (e.g. cardiovascular disease, diabetes) or other clinical motor features (e.g., MDS-UPDRS III, Hoehn and Yahr stage) also played a significant role in defining the resulting clusters. Among the domain-specific cognitive assessments, executive, memory and attention functions emerged as the most relevant, whereas language and visuospatial abilities had lower contributions.

### Diagnostic prediction strength comparison between the clinical diagnostic reference test and the optimal data-driven model

**Fig. 1:**
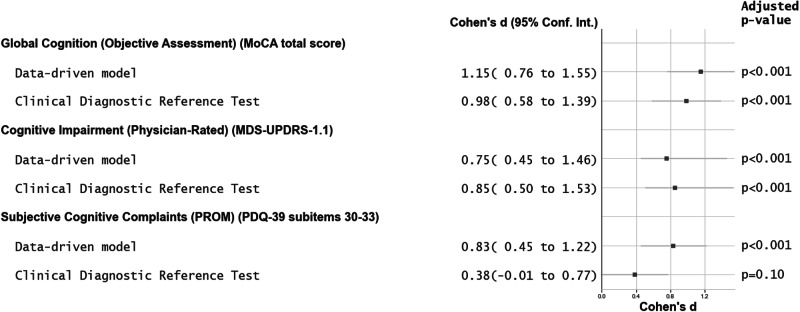
Diagnostic prediction strength. Clinical diagnostic reference test vs data-driven model. Forest plot displaying Cohen’s *d* effect sizes and 95% confidence intervals for various features assessed by the data-driven model and the clinical diagnostic reference test. A bootstrap approach was employed to assess the statistical significance, which was determined by p-values adjusted for multiple comparisons using the Benjamini–Hochberg method *p*. The alpha level was set at *α* = 0.05. The analysed features include the MoCA total score, MDS-UPDRS 1.1 and the sum of the PDQ-39 subitems 30–33.

The optimal data-driven model and the clinical diagnostic reference test were applied to the PD study population, and their diagnostic predictive strength was compared. The predictive performance of both diagnostic tools was evaluated based on their ability to maximise distinctions between PD-NC and PD-MCI phenotypes using a set of predefined measures of cognitive performance: an objective cognitive assessment measured by global cognition (MoCA total score), a physician-rated score of cognitive impairment (MDS-UPDRS 1.1) and a patient-reported outcome measure (PROM) measuring subjective cognitive complaints (Parkinson's Disease Questionnarie-39 (PDQ-39) subitems 30–33) hypothesised to differ between these groups. Overall, the effect sizes obtained by both diagnostic tools were comparable (Fig. 1). The data-driven model demonstrated higher effect sizes in global cognition (MoCA total score) and subjective cognitive complaints (PDQ-39 subitems 30–33) compared to the clinical diagnostic reference test, both of which were statistically significant (Fig. 1). Conversely, the clinical diagnostic reference test showed higher effect sizes in cognitive impairment rated by a physician (Fig. 1). A bootstrap approach was employed to assess the statistical significance of the differences in effect sizes across the set of clinical characteristics mentioned before in this subsection; however, no significant differences were observed. These results suggest that the data-driven model is non-inferior to the clinical diagnostic reference test, proving its ability for MCI diagnosis.

### Identification of cognitively distinct PD subgroups

After confirming the non-inferiority of the data-driven model compared to the clinical diagnostic reference test, a detailed analysis was conducted to explore the ability of the data-driven approach to identify MCI subgroups potentially overlooked by the clinical diagnostic reference test.

PwPD were categorised into four subgroups by comparing the overlap of the PD-NC and PD-MCI groups between the data-driven model and the clinical diagnostic reference test approach. This procedure resulted in the following groups: (1) NC group, participants with PD classified as NC by both diagnostic tools (*n* = 49); (2) Misclassified, participants with PD classified as NC by the data-driven model and as MCI by the clinical diagnostic reference test (*n* = 1); (3) Misclassified, participants with PD classified as MCI by the data-driven model and as NC by the clinical diagnostic reference test (*n* = 26); (4) MCI group, participants with PD classified as MCI by both diagnostic tools (*n* = 39). The group consisting of a single patient was excluded from the follow-up analysis due to its insufficient sample size for meaningful statistical comparisons.

**Fig. 2:**
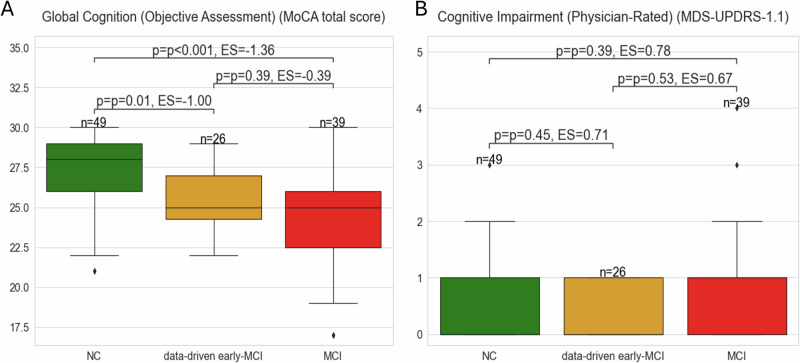
Validation of the data-driven model for MCI subgroup identification. The figure shows the comparison of the cognitive performance of the subgroups of participants with PD. The distribution of the global cognitive function (MoCA total score) and the physician-reported cognitive impairment status (MDS-UPDRS 1.1) among the different subgroups is displayed in Panel A and B, respectively. These variables were selected as they were not included in the model training, and are expected to differ between PD-NC and PD-MCI individuals. Statistical comparisons were performed using two-tailed Mann–Whitney U tests (due to the non-normal distribution of the features), with p-values adjusted for multiple comparisons using the Benjamini–Hochberg procedure. The alpha level was set at *α* = 0.05. Outliers are represented as individual diamonds, and the interquartile ranges are shown within each box.

External variables measuring cognitive function (objective cognitive assessment measuring global cognition (MoCA total score), and a physician-rated score of cognitive impairment (MDS-UPDRS 1.1)) were selected to investigate and determine the clinical phenotype of the data-driven early-MCI group (*n* = 26). Distributional analyses were conducted to quantify the differences between the groups at the baseline visit. The data-driven early-MCI group demonstrated statistically significant differences from the NC group in MoCA total scores, indicating poorer global cognitive function (Fig. 2A). These results, beyond showing a specific profile of cognitive impairment, constitute a clinical validation of the existence of the data-driven early-MCI group as a separate clinical entity from NC. However, no significant differences were observed for the MDS-UPDRS 1.1 due to the lack of sensitivity of the score (Fig. 2B).

**Fig. 3:**
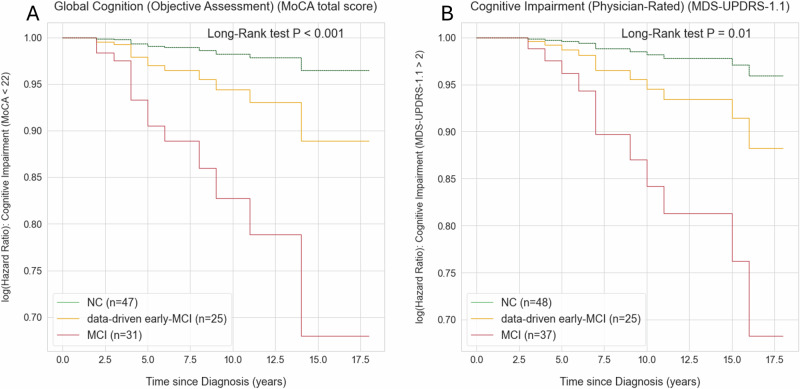
Validation of the data-driven model for MCI subgroup identification. The current figure illustrates the risk of reaching cognitive impairment that affects activity daily of living across the different cognitive groups. This cognitive impairment was defined as either **A**) a MoCA total score smaller or equal to 21 or **B**) a moderate score in MDS-UPDRS 1. Individuals that reached the endpoint at the baseline visit were excluded from this analysis, resulting in different numbers of individuals in each analysed feature. The *X*-axis represents time since diagnosis (in years), and the *Y*-axis shows the log (hazard ratio). Each subgroup is represented by a trajectory and colour: NC (green), data-driven early-MCI group (yellow), and MCI (red). The following confounders were included in the Cox hazard analyses: age, years of education and score of the feature at baseline. Sex was not included as confounder as it violated the proportional hazards assumption.

Moreover, Cox proportional hazards models were applied to investigate whether the subgroups displayed different longitudinal trajectories in converting to moderate cognitive impairment. The trajectory of the data-driven early-MCI group was distinct and differed statistically from both the MCI and NC groups in progressing towards moderate impairment, as measured by the MoCA total score (global cognition) and the MDS-UPDRS 1.1 (physician-rated cognitive impairment), with global log-rank test *p*-values of *p* < 0.001 and 0.01, respectively (Fig. 3). Post-hoc analyses were conducted to study the differences between the NC and the data-driven early-MCI group, and significant differences were found for MoCA total score (0.03) and MDS-UPDRS 1.1 (0.04).

### Cognitive characterisation of the identified groups

**Fig. 4:**
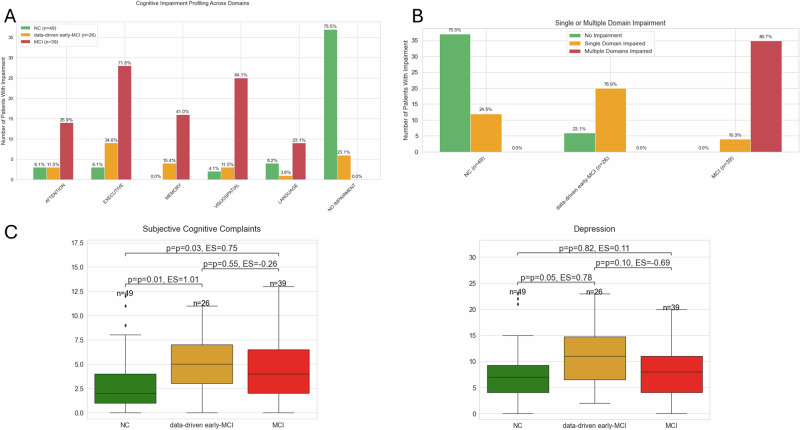
Cognitive impairment profile. **A** Bar plot showing the proportion of individuals within each group (NC: green, data-driven early-MCI: yellow, MCI: red) without any impairments or with an impairment by cognitive domain (attention, executive, memory, visuospatial, and language). **B** Bar plot showing the proportion of individuals without impairment (green), single domain impairment (yellow), and multiple domain impairment (red) within the NC, data-driven early-MCI, and MCI groups. Percentages, in both graphs, were calculated relative to the total number of participants in each group (NC: *n* = 49; data-driven early-MCI: *n* = 26; MCI: *n* = 39). **C** Box plot showing the differences in depression and subjective cognitive decline across the identified groups. Each subgroup is represented by a colour: NC (green), data-driven early-MCI group (yellow), and MCI (red). Statistical comparisons were performed using two-tailed Mann–Whitney U tests (due to the non-normal distribution of the features), with *p*-values adjusted for multiple comparisons using the Benjamini–Hochberg procedure. The alpha level was set at *α* = 0.05. Outliers are represented as individual diamonds, and the interquartile ranges are shown within each box.

After validating the existence of the data-driven early-MCI group as a distinct clinical entity, a detailed characterisation of the subgroups’ phenotype, at the baseline visit, was conducted (Table 2, Fig. 4 and Supplementary Table 3).

**Table 2:** Clinical and socio-demographic characterisation of the groups

	**NC group (*n*** = 49)	**Data-driven early-MCI group (*n*** = 26)	**MCI group (*n*** = 39)	**NC vs Data-driven early-MCI group**	**Data-driven early-MCI group vs MCI**	**NC vs MCI**
Features	Median (IQR)			Adjusted *p*-value		
Socio-demographic						
Age	61.58 (13.44)	69.37 (17.60)	69.14 (14)	0.14	0.93	0.014
Years of education	14.0 (5.0)	13.0 (5.0)	13.0 (5.5)	0.53	0.93	0.38
Clinical						
Cognitive performance						
TMT-A (seconds)	36.0 (16.0)	51.0 (17.5)	50.0 (27.0)	*p* < 0.001	0.85	*p* < 0.001
Block span (Total score)	8.0 (2.0)	7.0 (2.0)	7.0 (3.0)	0.10	0.24	*p* < 0.001
TMT(B-A) (seconds)	37.0 (19.0)	62.5 (45.5)	81.0 (58.5)	*p* < 0.001	0.22	*p* < 0.001
FAB	17.0 (2.0)	15.0 (2.5)	14.0 (3.0)	0.002	0.057	*p* < 0.001
Word list learning total score	25.0 (3.0)	20.5 (4.0)	19.0 (5.0)	*p* < 0.001	0.56	*p* < 0.001
Word list delayed recall	8.0 (2.0)	5.0 (2.75)	6.0 (4.0)	*p* < 0.001	0.52	*p* < 0.001
Judgement of line orientation	26.0 (4.0)	24.5 (6.5)	23.0 (7.5)	0.70	0.032	0.002
MoCA clock	3.0 (0.0)	3.0 (0.0)	2.0 (1.0)	0.36	0.08	*p* < 0.001
Phonemic fluency S	10.0 (5.0)	8.0 (6.5)	7.0 (6.0)	0.78	0.45	0.22
MoCA naming	3.0 (0.0)	3.0 (0.0)	3.0 (0.0)	0.97	0.53	0.36
Motor						
Hoehn and Yahr	2.0 (0.5)	2.0 (0.375)	2.0 (0.0)	0.046	0.55	0.36
MDS-UPDRS III	23.0 (19.0)	28.5 (18.75)	30.0 (15.0)	0.27	0.65	0.042
ADL						
PDQ-39 subitems 11–16	5.0 (7.0)	5.5 (6.0)	4.0 (7.5)	0.88	0.97	0.91
Neuropsychiatric symptoms						
Depression (BDI-I)	7.0 (5.25)	11.0 (8.25)	8.0 (7.0)	0.048	0.075	0.80
Apathy (SAS)	11.0 (5.0)	15.5 (6.75)	13.0 (5.5)	0.008	0.23	0.15
Subjective Cognitive Decline						
Subjective Cognitive Complaints (PDQ-39 subitems 30-33)	2.0 (3.0)	5.0 (4.0)	4.0 (4.5)	0.01	0.55	0.033
Disease-related factor						
Disease duration	2.0 (4.0)	3.5 (5.0)	3.0 (7.0)	0.22	0.66	0.46
Age at PD onset	58.0 (15.0)	62.0 (19.75)	64.0 (13.0)	0.41	0.82	0.042

Descriptive statistics are reported with median and interquartile range (IQR). Distributional analyses were conducted using the two-tailed *t*-test for numerical variables (normally distributed), two-tailed Mann–Whitney U test for numerical variables (not normally distributed) and Pearson’s chi-squared test for categorical variables. Statistical significance was determined by *p*-values adjusted for multiple comparisons using the Benjamini-Hochberg method (adj. *p*-value). The alpha level was set at *α* = 0.05. Significant *p*-values were highlighted in bold. Abbreviations: *MoCA* Montreal Cognitive Assessment, *TMT* Trail Making Test, *ADL* Activities of Daily Living, *FAB* Frontal Assessment Battery, *PDQ-39* Parkinson's Disease Questionnaire, *BDI-I* Beck Depression Inventory, *SAS* Starkstein Apathy Scale, *MDS-UPDRS* Movement Disorder Society-Unified Parkinson’s Disease Rating Scale.

The NC group (*n* = 49) presents low impairment, with no amnestic nor multidomain impairment, where over 75% of individuals (*n* = 37) do not show any impairment (Fig. 4). Within the data-driven early-MCI group (*n* = 26), 23% of the individuals (*n* = 6) did not present any cognitive impairment and the remaining ones (*n* = 20) present a single domain impairment profile where non-amnestic impairment dominates, with memory being impaired in 15.4% of the individuals (*n* = 4). The most impaired domain was executive function (*n* = 9, 34.6%) followed by memory (*n* = 4, 15.4%), visuospatial (*n* = 3, 11.5%) and attention (*n* = 3, 11.5%). On the other hand, the MCI group presents a completely distinct cognitive impairment profile, with all individuals presenting an impairment in at least one domain and the prevalence of multi-domain impairment being higher than 90% (Fig. 4). The most impaired domains were executive function and visuospatial abilities, affecting over 72% (*n* = 28) and 64% (*n* = 25) of individuals, respectively (Fig. 4). Amnestic impairment (memory) affected 41% of the MCI individuals (*n* = 16). A more detailed characterisation of the individual cognitive impairment profiles is shown in Supplementary Fig. 2.

Looking at the different neuropsychological assessments between the data-driven early-MCI and the NC group, a heterogeneous pattern of cognitive impairment was found in the data-driven early-MCI group. Statistically significant differences were observed in attention Trail Making Test Part A (TMT-A), executive function Trail Making Test Part B minus Part A (TMTB-A) and Frontal Assessment Battery (FAB) and verbal memory (Word list learning total score, Word list Delayed Recall), where the misclassified group showed a higher cognitive impairment in these domains when compared to the NC group. No differences were found in visuospatial abilities (judgement of line orientation, MoCA clock) or language (phonemic fluency S, MoCA naming) (Table 2). Moreover, additional statistically significant differences on other clinical characteristics were found, such as disease stage (Hoehn and Yahr scale), severity of neuropsychiatric symptoms (depression and apathy) and subjective cognitive complaints (PDQ-39 subitems 30–33), indicating a worsening of the condition of the data-driven early-MCI group compared to the NC group. However, no differences were observed on motor function (MDS-UPDRS III), ADL or disease-related factors such as disease duration or age at PD onset (Table 2 and Fig. 4). Apart from initially detected differences in age, which were no longer significant after adjustment, no major differences were found in socio-demographic variables (Table 2).

These results suggest that the data-driven early-MCI group has a higher cognitive impairment profile when compared to the NC group in attention, memory and executive functions and may be a distinct clinical entity.

## Discussion

In this study we developed and tested a new data-driven diagnostic tool for the early detection of cognitive impairment by targeting MCI in PwPD. By leveraging different sources of clinical data covering a comprehensive neuropsychological test battery, motor and staging characteristics (e.g., MDS-UPDRS III, Hoehn and Yahr stage), disease-related factors, comorbidities and clustering methodologies combined with different domain weighting, the developed model demonstrated non-inferiority to the clinical diagnostic reference test (MDS PD-MCI Level II) in its ability to produce group classifications that showed comparable effect size differences.

An intriguing finding of our diagnostic approach was the ability of the data-driven model to identify subgroups of MCI with a mild impairment pattern, which was validated by a longitudinal analysis. Interestingly, this subgroup cannot be captured by the clinical gold standard (MDS PD-MCI Level II). Here, the data-driven model classified patients with NC as MCI patients. This data-driven early-MCI group differed from the NC group in terms of global cognition, executive memory and attention domains but showed milder levels of cognitive impairment than the MCI group. Importantly, analyses on the follow-up visit data revealed that this data-driven early-MCI group displays an intermediate cognitive progression trajectory in terms of global cognition (MoCA total score) and physician-rated cognitive impairment (MDS-UPDRS 1.1). Interestingly, no significant differences were observed in physician-rated cognitive impairment (MDS-UPDRS 1.1) at the baseline visit, where both true NC and data-driven detected MCI patients were similarly rated by these physicians. These findings suggests that the group identified by the data-driven model is not a misclassified group, but a clinically distinct profile detected by the data-driven model, that extends beyond cognitive decline related to ageing (NC), the clinical staging interpretation remains ambiguous as the mild profile observed in these patients could reflect either an early or subtle mild cognitive impairment phenotype. In the comparison of multi-domain versus single-domain impairment, the MCI group (*n* = 39) showed a high prevalence of multi-domain impairment, affecting 90% of individuals (*n* = 35). In contrast, in the data-driven early-MCI group (*n* = 27), single-domain impairment predominated, observed in 77% of individuals (*n* = 21). For the NC group (*n* = 49), the majority of participants – 75% (*n* = 37) – showed no cognitive impairment. Although the proportion of multi-domain impairment on the MCI group might be a bit higher than expected, several studies reported similar or comparable results when applying MDS PD-MCI Level II criteria with a 1.5 SD threshold, such as Marras et al. and Goldman et al., reporting 93% of multiple-domain impairment within the MCI group and Broeders et al. reporting lower percentages (65%) but still comparable to our findings^21,29,30^.

The developed data-driven diagnostic tool offers a novel approach to diagnosing MCI by emphasising the most relevant factors in the clustering process. Specifically, domain-specific cognitive assessments emerged as the primary drivers in the data-driven model. Additionally, disease-related factors (e.g., age at diagnosis, disease duration), comorbidities (e.g., cardiovascular disease, diabetes), and other clinical characteristics (e.g., MDS-UPDRS III, Hoehn and Yahr stage) played a significant role in defining the resulting clusters. These findings underscore the relevance of non-cognitive factors, including comorbidities, disease-related factors, and clinical characteristics, in MCI diagnosis and specifically in the identification of subgroups overlooked by the clinical diagnostic test. The model’s strength lies in its heightened sensitivity to specific cognitive domains, such as memory, executive function, and attention. In contrast, the clinical diagnostic reference test assigns equal weight to all cognitive domains^16^. The absence of predefined cutoffs in the data-driven model, combined with its domain-specific weighting, allowed a different perspective, making it a comprehensive tool in terms of MCI diagnostic. Within the domain-specific cognitive assessments, the most important features for the data-driven model were Word list Delayed Recall (memory function), TMT B-A (executive function), Word list learning total score (memory function), and FAB (executive function).

Diving into the clinical staging more thoroughly, we analysed follow-up data from both the MoCA total score and the MDS-UPDRS 1.1. These analyses revealed distinct but stable cognitive trajectories across the three groups. The data-driven group that was detected as having early or subtle mild cognitive impairment, exhibited consistently a milder impairment profile compared to the MCI group, but higher than the NC one. The progression trajectory did not overlap with the one of the NC group, nor did it converge with the more pronounced decline seen in the MCI group. This pattern suggests that the data-driven model may have detected a group that corresponds to an early or subtle mild cognitive impairment group.

The analysis conducted at the baseline visit provided additional insights regarding the clinical staging interpretation further supporting the early-MCI stage rather than the subtle mild cognitive impairment one. The predominance of executive impairment, alongside deficits in working memory and attention, commonly referred to as a frontal cognitive impairment phenotype^31^, is consistent with cognitive profiles observed in early-stage MCI individuals in the literature^32,33^. Notably, deficits in mental flexibility (executive function) and working memory (attention) are among the earliest cognitive domains affected^32,33^. In contrast, visuospatial deficits were less pronounced in this group, which aligns with the expected clinical picture in the earliest phases of MCI, as they are usually presented in more advanced stages of MCI. Meanwhile, individuals in the MCI group showed more substantial visuospatial impairments, as anticipated from the literature^32,33^. Moreover, individuals in the data-driven MCI group showed significantly higher levels of self-perceived cognitive deficits (subjective cognitive complaints), as well as more severe depressive and apathy symptoms (Fig. 4 and Table 2). Both depressive and apathy symptoms are well-established risk factors for the subsequent development of MCI and dementia, which can be indicative of an early MCI profile^34–36^. Notably, depressive symptoms are known to influence both subjective cognitive complaints and frontal-executive cognitive impairment profiles^35–37^. While this group shows subjective cognitive complaints and mild objective impairments, its clinical interpretation remains challenging. On one hand, the presence of clear subjective complaints could suggest a link with subjective cognitive decline (SCD), a well-established pre-MCI risk state in Alzheimer’s disease. However, the lack of consensus and formal guidelines for defining SCD in Parkinson’s disease leads to high heterogeneity across people with PD-SCD, making characterisation in this population challenging^38,39^. SCD has been conceptualised largely in the Alzheimer’s field, if the SCD definition proposed by the subjective cognitive decline initiative (SCD-I) in Alzheimer’s disease would be used as a proxy, most individuals in this group would not meet the criteria as they already present measurable deficits in executive function, attention, and working memory^40^, which in combination with elevated depressive and apathy symptoms may place them beyond a purely preclinical stage of SCD and closer to an early or mild cognitive impairment phenotype.

This raises interesting conceptual and practical questions: does this group reflect individuals on the cusp of MCI, where subjective concerns and psychiatric symptoms act as early markers of future progression, or does it constitute a mild but stable MCI subtype, particularly sensitive to executive dysfunction. The longitudinal analyses suggest that these patients do not follow the same trajectory as cognitively normal individuals, indicating that this is not merely a subjective or psychiatric phenomenon, but instead a subtle, quantifiable decline with clinical relevance. Nevertheless, more extended follow-up is required to determine whether this profile represents a stable, mild endophenotype of MCI or a dynamic pre-MCI stage progressing toward more typical MCI presentations.

Altogether, the identification of a group overlooked by the clinical diagnostic reference test underscores the potential of data-driven approaches to enhance and complement clinical decision-making by identifying subgroups and subtle cognitive changes. The mechanisms by which the data-driven model identified this novel group provide valuable insights and a foundation for future studies aimed at improving MCI diagnosis, with a focus on developing abbreviated data-driven models prioritising the study of memory and executive functions. Such models could facilitate the creation of MCI diagnostic tools that are more generalisable and applicable in routine clinical care^28^.

This study has some methodological limitations that should be acknowledged. A key limitation is the lack of clinical validation of the cognitive status of the individuals from the standard of care physician, which complicates the benchmarking and validation of the data-driven model in terms of accuracy and precision. Also, it should be noted that MDS-UPDRS 1.1 and PDQ-39 subitems used through all the analyses are short and not comprehensive cognitive scales. In particular, the PDQ-39, when used to assess ADL impairment in PwPD, has limitations due to its nature as a self-reported outcome, as patients with dementia may not provide fully reliable information. However, in our cohort, the PDQ-39 was the most widely available ADL assessment. While other informant-based tools, such as the Functional Activities Questionnaire (FAQ), were introduced later in the study, their data coverage was more limited.

In addition, the lack of normative values specific to the Luxembourgish population posed a challenge. To address this, statistical procedures based on regression analyses were applied to estimate normative data from the healthy control group. Although this approach has been proposed and used in the literature, it is less precise than established normative values, which are typically derived from larger and more representative samples. As a result, this limitation, combined with the reduced number of available healthy controls, may introduce variability in the calculation of z-scores and the identification of cognitive impairments. An additional source of variability in the z-score calculations arises from the fact that some individuals performed the tests in a non-native language due to the linguistic diversity of Luxembourg’s population. Another important consideration is the lack of universal consensus on how to group neuropsychological tests into cognitive domains. Different grouping strategies may yield varying results, potentially influencing the interpretation of domain-specific impairments. Additionally, the selection of participants for the extensive neuropsychological assessment represents a potential source of bias, as it was performed on a voluntary basis rather than systematically across the cohort.

In applying the MDS PD-MCI Level II criteria, the choice of the 1.5 SD threshold to define cognitive impairment is a critical factor that may introduce heterogeneity in the diagnosis of MCI. Alternative thresholds, such as 1 SD or 2 SD, could yield slightly different outcomes. However, consistent with the recommendations of Dalrymple-Alford et al.^22^, we considered that 1.5 SD provides the most suitable framework for MCI detection. Another limitation is that, while we followed the cognitive testing–based inclusion criteria for PD-MCI diagnosis, we were unable to fully apply the broader PD-MCI criteria proposed by Litvan et al.^16^, which require documentation of gradual cognitive decline and preserved ADL, due to the lack of consistent longitudinal data and detailed informant-based ADL measures across all participants.

Lastly, the effects of levodopa and other dopaminergic drugs on cognitive performance were not accounted for in this study. While these medications play a crucial role in alleviating and controlling motor symptoms in PwPD, their impact on cognition remains unclear and mixed. In some individuals, they may enhance executive function, whereas in others, they can contribute to impulse control disorders, learning impairments, or even psychosis^41–43^. Other aspects, such as genetic factors, were not considered.

Besides clinical or patient-specific characteristics, some methodological limitations should be acknowledged, such as the lack of external validation for the data-driven model. Although the results have clinical validation, successful replication in independent cohorts would further ensure the absence of overfitting and strengthen methodological confidence in the findings. Moreover, the relatively small sample size (*n* = 116) compared to the number of input features (*n* = 21) may increase the risk of overfitting and introduce potential bias into the model. Further research is needed to validate the data-driven model in independent cohorts, as well as to conduct feasibility studies assessing the potential applicability of the screening model in routine clinical practice by evaluating factors such as data availability, usability, and resources required for implementation. Finally, further research is also warranted to directly compare the data-driven and cognitive testing–based models with the full gold-standard clinical assessment in a prospectively designed study including detailed longitudinal and informant-based ADL data.

We demonstrate how a data-driven model can provide with a different perspective in the diagnosis of MCI allowing to identify early patterns of impairment. Through the comparison with the standard clinical assessment test (MDS PD-MCI Level II), a unique clinically distinct subgroup has been identified, whose subtle cognitive changes may be overlooked by the traditional criteria. This allows for early identification of patients with a higher risk, thereby facilitating early interventions that can slow down the progression to dementia.

## Methods

### Study population

The Luxembourg Parkinson’s study (LuxPark) is a prospective longitudinal cohort of individuals with Parkinsonism, including more than 850 idiopathic and atypical individuals, and around 900 healthy control participants^44,45^. All the subjects have signed a written informed consent, and the collection has been approved by the National Ethics Board (CNER Ref: 201407/13) and Data Protection Committee (CNPD Ref: 446/2017). PwPD were followed annually for up to 8 years while healthy controls had one follow-up visit after 4 years. For the current analysis, only participants with idiopathic PD, meeting the inclusion criteria proposed by the United Kingdom Parkinson’s Disease Society Brain Bank Clinical Diagnostic Criteria^46^ (*n* = 736) and with complete in-depth neuropsychological (NPSY) assessments at the baseline visit (*n* = 196) were selected. The primary reason for this study population reduction is the availability of neuropsychological assessment data. Within the LuxPark study, two levels of comprehensiveness were used for the assessments, with the more extensive evaluation being optional and therefore conducted in fewer patients. Since the application of MDS PD-MCI Level II criteria requires the highest level of assessment, the number of eligible PD patients and healthy controls was considerably reduced. To ensure that cognitive function was not influenced by neurological or psychiatric conditions unrelated to PD, participants with a score higher or equal than 30 out of 63 in the Beck Depression Inventory-I (BDI-I)^47^, diagnosed with bipolar disorder or schizophrenia were excluded. Additionally, participants with self-reported seizures, strokes, traumatic brain injuries (TBI) or brain tumours were excluded. Furthermore, healthy control participants at risk of developing PD, defined by a family history of PD or the presence of risk factors such as Rapid Eye Movement (REM) sleep behaviour disorder (RBD), defined by a score ≥ 6 in the REM Sleep Behaviour Disorder Screening Questionnaire (RBDSQ)^48^, diabetes, alcohol consumption, smoking, hypertension and cardiovascular disease, were excluded from the analysis. Healthy control participants presenting any psychiatric or neurological condition mentioned above were also removed. Lastly, participants with Parkinson’s disease dementia (PDD) were excluded. This was determined using the criteria proposed by Dubois et al.^49^, which require that Parkinson’s disease precedes the onset of dementia, that there is global cognitive impairment defined as an MMSE score below 26 (corresponding to a MoCA score below 22)^50^, and that dementia has a significant impact on ADL, operationalized as a score greater than 14 on the ADL subscore of the PDQ-39. In addition, participants were required to have no evidence of major depression (BDI-I score ≥30)^47^ and absence of delirium. The application of the inclusion and exclusion criteria resulted in a dataset of 226 healthy controls and 115 PwPD constituting the study population for the analyses presented in this paper. Follow-up visits from this study population were also available for conducting further meta-analyses.

### Overview of available data

Data collected from participants included socio-demographic characteristics, clinical history, details of medication use, results of clinical examinations, PROMs and clinician-reported outcome measures (ClinRO), as well as other relevant disease-related factors. Socio-demographic variables comprised age, sex, years of education. The clinical history section covered past diagnoses, such as diabetes and hypertension, as well as lifestyle factors that are potential risk factors, such as smoking and alcohol consumption, and other disease-related factors, such as age at PD onset and disease duration. PROMs were collected through psychometric scales and quality of life surveys. Depressive symptoms were assessed using the BDI-I^47^, and apathy using the Starkstein Apathy Scale (SAS)^51^. ADL were measured using the sum of PDQ-39 subitems 11 to 16^52^, and subjective cognitive complaints using the sum of PDQ-39 subitems 30 to 33. ClinRO measured cognitive impairment in patients. Examination data included assessments of motor function using the MDS-UPDRS-III^53^, as well as assessments of cognition focusing on both global cognition and specific cognitive domains. Global cognitive functioning was measured using the MoCA^11^. A comprehensive neuropsychological test battery was also administered to evaluate performance across the five main cognitive domains: attention, executive function, memory, visuospatial abilities, and language, further details on the test selection is provided in the 'Neuropsychological test selection' subsection. Additional information on the implemented tests can be found in Hipp et al. (2018)^44^.

### Defining PD-MCI and cognitive domain impairment

In the present study, PD-MCI was defined according to the MDS PD-MCI criteria^16^. As mentioned in the introduction, this definition is divided into two levels of comprehensiveness: (a) Level I (abbreviated assessment), where only one test per cognitive domain is used and (b) Level II (comprehensive assessment), which involves the usage of two tests per domain. Consequently, the definition of PD-MCI varies across levels. While both levels require impairment in at least two tests, the interpretation of these impairments differs. In Level I, a patient must show impairment in at least two cognitive domains, whereas in Level II, impairment in only one domain (assessed by 2 independent tests) is sufficient to diagnose PD-MCI. Another distinction is that in Level I, individuals must perform within the normative threshold in 80% of the tests (4 out of 5) to avoid classification as MCI, whereas in Level II, this percentage increases to 90% (9 out of 10)^16^. In our study, a PwPD was classified as having PD-MCI if impairment was observed in at least two tests, regardless of whether the NPSY tests assessed the same domain or different domains following the MDS PD-MCI level II criteria^20^. Reduced performance on an NPSY test was defined as a score of 1.5 SD or more below the age-, sex-, and years of education adjusted normative mean further details on how the z-scores are defined are given in the 'Normative data calculation' subsection. This threshold was chosen as it provides a suitable balanced criteria, avoiding an excess of false positives associated with less conservative thresholds (e.g., 1 SD) while also minimising false negatives linked to more restrictive thresholds (e.g., 2 SD)^16,22^.

### Neuropsychological test selection

In cases where multiple tests were available for a given domain, the selection was based on three main factors: (1) to maximise the number of participants included in the study by giving priority to tests that have been carried out on a maximum number of subjects, (2) using current knowledge on which specific tests provide better accuracy for assessing MCI in PwPD^21^, and (3) the expertise of neuropsychologists in allocating specific features to each domain, since most tests cover several domains.

For the current analysis, two tests were selected for each of the five major cognitive domains. Attention was assessed using the TMT-A^54^ and the Block-Tapping test (Forward)^55^. Executive function was evaluated using the FAB^56^ and the TMT B-A^54^. Verbal episodic memory performance was measured using the Word list learning total score and Word list Delayed Recall from the word list memory subset from the Consortium to Establish a Registry for Alzheimer’s Disease (CERAD) (RRID:SCR_003016) battery^57^. Visuospatial abilities were assessed with the Judgement of Line Orientation test^58^ and the Clock Drawing Test, part of the MoCA assessment^11^. Finally, language was evaluated using the Verbal Fluency Test (phonemic) and the Naming sub-item within the MoCA^11^.

### Normative data calculation

The definition of impairment is based on z-scores, which represent how many SDs an individual’s test performance deviates from the population mean after adjusting for relevant confounders. Therefore, these z-scores are critical to the implementation of the MDS PD-MCI Level II criteria, whose calculation relies on the availability of normative data for the given population.

As population-specific normative data are not available for Luxembourg's population, normative values (expected scores for non-PD individuals) were derived from the healthy control subgroup included in this study, following an approach previously described in the literature^59^. In this approach, the normative values are the expected values in an NPSY test for a non-PD patient adjusted to their socio-demographic characteristics (age, sex, and years of education). These socio-demographic features are commonly considered confounding variables in NPSY assessments because they represent the most relevant non-pathological factors that can significantly influence cognitive performance. The approach outlined in Shirk et al.^59^, is claimed to effectively account for the effect of confounders on cognitive ability by using multivariate linear regression and has been applied by other studies^60–62^. In this regression, the outcome variable (*Y*_obs_) is the observed cognitive test score, and the covariates or regressors are the confounders (sex, age, years of education). This method yields coefficients that quantify the amount of change in the outcome variable per unit change in the covariate, while controlling for all other confounders. These coefficients, together with the intercept, are used to calculate the normative values according to Equation 1. The intercept is modified by adding or subtracting the effect of the confounders, calculated as the product between the coefficient and the individual’s confounding variable, resulting in the expected value (*Y*_exp_) adjusted specifically to the age, sex, and education of the individual. Once the normative value is obtained, Equation 2 is applied to calculate the z-scores. An overview of the z-score distributions is presented in Supplementary Fig. 1.

Equation 1

$${\rm{B}})$$

Where:

*Y: Intercept of the linear regression*

${\boldsymbol{f}}{\boldsymbol{age}}$ *: Coefficient for the age variable*

*Age:    Age of the individual*

${\boldsymbol{f}}{\boldsymbol{ed}}$ *: Coefficient for the education level variable*

*ed:    Years of education of the individual*

${\boldsymbol{f}}{\boldsymbol{sex}}$ *: Sex coefficient for the sex variable*

*Sex:    Sex of the individual*

The calculation of z-scores is based on Equation 2.

Equation 2

$${\boldsymbol{Z}}=\frac{{{\boldsymbol{Y}}}_{{obs}}-{{\boldsymbol{Y}}}_{\exp }}{{\boldsymbol{SD}}}$$

Where:

**Z**:    Z-score of a cognitive test of an individual

**Y**_obs_:   Individual's observed score on a given cognitive test

**Y**_exp_:   Expected (predicted) population mean score (normative value)

**SD**: Standard deviation from the normative data

### Data-driven model

Unsupervised learning approaches, specifically clustering algorithms, were employed. Three clustering algorithms were evaluated: SC using symmetric normalised Laplacian, due to its highest robustness and Euclidean distance for constructing the affinity matrix; K-Means, and GMM.

The input data for the MCI diagnostic model consisted of the selected NPSY assessments encompassing all five major cognitive domains at baseline visit, described in the 'Neuropsychological test selection' subsection of the methodology. Disease-related factors, such as age at diagnosis and disease duration, were also considered. Additionally, well-established self-reported risk factors, such as comorbidities including cardiovascular disease, hypertension, and diabetes, and lifestyle factors such as smoking and alcohol consumption^63^, were included in the analysis. Finally, other variables describing the progression of PD were also included: motor symptoms (MDS-UPDRS III), disease stage (Hoehn and Yahr), and RBDSQ^48^. For this analysis a cross-sectional dataset covering the baseline visit was used.

Before training, input features underwent tailored preprocessing to enhance model performance. Categorical variables, such as risk factors, were encoded numerically using a one-hot encoder, while numerical variables were normalised using robust scaling. NPSY test scores, already standardised as *Z*-scores, required no additional encoding. The TMT B-A and TMT-A scores were inverted so that lower scores corresponded to worse performance, aligning with the interpretation of other neuropsychological measures where lower scores indicate poorer performance.

To optimise the clustering performance, a grid search was used to find the optimal set of hyperparameters for the distinct clustering algorithms. Hyperparameter optimisation was guided by both internal and external criteria. Internal validation focused on cohesion and separation metrics, which assess within-cluster similarity and between-cluster distinctiveness^64^. To conduct the internal validation, the silhouette score^65^, a widely adopted internal clustering metric, was used to get the best hyperparameters for each of the clustering trains. This metric provides normalized scores and helps mitigate cluster size-related bias^66^.

Given the objective of distinguishing between PD-NC and PD-MCI, clinical assumptions regarding the phenotypes of these groups were incorporated (external criteria). These rules assumed that individuals with PD-MCI would be older, have a longer disease duration, and advanced Hoehn and Yahr stages, and would have lower MoCA total scores^67^. Clustering results inconsistent with these clinical expectations were excluded from further analysis.

Aligning with the purpose of distinguishing between PD-NC and PD-MCI, the number of clusters was set to two. Three or four clusters were also tested, but two clusters remained the most optimal solution (Supplementary Table 1). For enhancing interpretability, feature importance was assessed by examining the contribution of each input variable to the clustering by using the mutual information score.

A global comparison between the best models from the different algorithms was conducted by evaluating reported metrics, such as accuracy, sensitivity, and AUC in relation to the clinical diagnostic reference test (MDS PD-MCI Level II).

### Comparison of the diagnostic strength between the clinical diagnostic reference test and the best performing data-driven model

The diagnostic strength of the clinical diagnostic reference test (MDS PD-MCI Level II), and the best performing data-driven model were assessed by evaluating their ability to maximise distinctions between PD-NC and PD-MCI phenotypes based on a set of clinical characteristics hypothesised to differ between these groups^67^: an objective cognitive assessment, MoCA total score, which evaluates global cognition; a physician-rated score, MDS-UPDRS 1.1, which evaluates cognitive impairment; and a PROM, defined as the sum of PDQ-39 subitems 30 to 33, which evaluates subjective cognitive complaints.

The effect sizes between procedures were compared using a bootstrap resampling approach with 10,000 iterations. In each iteration, data for both groups (NC and MCI) and both procedures were resampled with replacement. Cohen’s *d* was computed for each procedure, and the difference between the effect sizes was stored. The 95% confidence interval of the bootstrapped differences was estimated from the 2.5^th^ and 97.5^th^ percentiles. A confidence interval including zero indicated a non-significant difference between effect sizes.

### Identification and characterisation of cognitively distinct subgroups

Cognitively distinct subgroups were identified by comparing how the PD-NC and PD-MCI groups overlap between the data-driven model and the clinical diagnostic reference test, and by running sub-analyses on the matched and misclassified groups resulting from this comparison.

Different analyses were conducted, both at the baseline visit (cross-sectional data) and follow-up visits (longitudinal data), using the MoCA total score (global cognition) and the MDS-UPDRS 1.1 (physician-rated cognitive impairment), to investigate the cognitive profile of the subgroups and validate the existence of the identified subgroups as distinct clinical entities.

Distributional analyses at the baseline visit (cross-sectional data) were conducted by selecting the most appropriate statistical method based on the feature distribution, while the statistical significance and effect sizes were reported as indicated in the 'Statistical analyses' subsection.

A longitudinal data analysis using Cox proportional-hazards models, which includes up to 8 visits of the whole study population, was conducted to assess significant differences in cognitive decline trajectories among the subgroups. For the Cox proportional hazards model, it was necessary to define a specific event or threshold in each outcome variable. In each case, the event was chosen to reflect the progression of individuals toward a level of cognitive impairment that significantly affects activities of daily living, beyond mild deficits. For MoCA (global cognition), a score of ≤21 was used^49,50^, indicating decreased global cognitive efficiency, while for MDS-UPDRS item 1.1 (physician-rated cognitive impairment), a score of ≥3 was applied, based on the established clinical interpretation of the subitem. We adopted a more conservative threshold, following Dubois et al.^49^, who defined decreased global cognitive efficiency as a score of <26. As MMSE data were not collected in this study, MoCA–MMSE conversion studies were used to guide the selection of the cut-off of MoCA ≤21, as a MMSE score of 26 corresponds to a MoCA score of 22. Individuals that already reached the endpoint at the first visit were excluded from this analysis due to the impossibility of determining at what time point the threshold was reached. A total of four PwPD died before experiencing the event of interest. Given the small number, and to preserve statistical power, these cases were treated as right-censored at the time of death, rather than modelled as competing events. Hazard ratios (HR) and 95% confidence intervals (CI) were calculated, and comparisons across groups were conducted using the global log-rank test. Each Cox model was adjusted for age, baseline score of the cognitive variable, and years of education. Sex was excluded from the analysis due to its violation of the proportional hazard assumption.

A detailed analysis of the group characteristics was conducted at the baseline visit to identify key differences across the socio-demographic and clinical profile (e.g. cognitive and motor function (MDS-UPDRS III), and other disease-related factors (age at diagnosis, disease duration)), see 'Statistical analyses' subsection for additional details on the statistical methods employed.

### Statistical analyses

Distributional analyses were performed using the two-tailed *t*-test for numerical variables (normally distributed), two-tailed Mann–Whitney U test was applied to numerical non-normally distributed features^68^, while categorical and ordinal data were analysed using the chi-squared test^69^. Statistical significance was reported using *p*-values, selecting an alpha level of *α* = 0.05, and effect sizes were calculated to quantify the magnitude of the observed differences. Cohen’s *d* was used for *t*-test; Standardised Point biserial correlation coefficient was used for Mann–Whitney U test, and Phi and Cramer’s V were used for Pearson’s chi-squared, Phi being specific for 2 × 2 contingency tables^70^.

Ordinal variables included the Hoehn and Yahr stage and the physician-rated cognitive impairment score (MDS-UPDRS 1.1). Categorical variables consisted of sex. Numerical variables not normally distributed included measures such as the TMT-A, Block Span, TMT B-A, FAB, Word list learning total score, Word list delayed recall, Judgement of Line Orientation, MoCA clock drawing, Phonemic fluency and naming (MoCA subitem), MoCA total score, disease duration, and activities of daily living (PDQ-39 subitems 11–16). Lastly, normally distributed numerical variables included years of education, MDS-UPDRS III, age, and age at PD onset. Normality of the features was checked using Shapiro–Wilk test.

All statistical analyses and machine learning models were performed using Python (version 3.10.7). All clustering methods were implemented using Scikit-learn version 1.2.1.

## Supplementary information

Supplementary Information

## Data Availability

Patient data used in the preparation of this manuscript were obtained from the National Centre of Excellence in Research on Parkinson’s Disease (NCER-PD). NCER-PD datasets are not publicly available, as they are linked to the Luxembourg Parkinson’s Study and its internal regulations. The NCER-PD Consortium is willing to share its available data. Its access policy was devised based on the study ethics documents, including the informed consent form, as approved by National Ethics Board (CNER Ref: 201407/13) and Data Protection Committee (CNPD Ref: 446/2017). Requests to access datasets should be directed to the Data and Sample Access Committee via email: [request.ncer-pd@uni.lu](mailto:request.ncer-pd@uni.lu).

## Code availability

The underlying code for this study is available on Gabriel Martinez / Data driven model for MCI screening · GitLab.
