# Supplementary material for: Data-driven clinical decision support tool for diagnosing mild cognitive impairment in Parkinson’s disease
Source: NPJ Parkinsons Dis. 2026 Jan 12;12:15. doi: 10.1038/s41531-025-01222-6 (PMC12800173; doi:10.1038/s41531-025-01222-6)
Supplement: Supplementary file 1 — Supplementary Information [file 41531_2025_1222_MOESM1_ESM.docx]

A data-driven clinical decision support tool for the diagnosis of Mild Cognitive Impairment in People with Parkinsons disease

**Gabriel Martínez Tirado^(1)^, Patricia Martins Conde^(1)^, Stefano Sapienza^(1)^, Holger Fröhlich^(2,3)^, Claire Pauly^(4,5)^, Valerie E. Schröder^(1,4,5)^, Sonja Jónsdóttir^(5)^, Olena Tsurkalenko^(1,4,5)^, Rejko Kruger^(1,4,5)^, Jochen Klucken^(1,4)^ on behalf of the NCER-PD consortium.**

1. *Luxembourg Centre for Systems Biomedicine (LCSB), University of Luxembourg, Esch-sur-Alzette, Luxembourg*
2. Bonn-Aachen International Center for IT, University of Bonn (b-it)
3. Fraunhofer Institute for Algorithms and Scientific Computing (SCAI)
4. *Centre Hospitalier de Luxembourg, Strassen, Luxembourg*
5. *Luxembourg Institute of Health (LIH), Strassen, Luxembourg*

# **Supplementary Information**

[Supplementary Panel 1. Z-score distribution of neuropsychological assessments within the PD and control group 2](#_Toc202350654)

[Supplementary Figure 1. Cognitive characterization and impairment profiles across the different PD subgroups 4](#_Toc202350655)

[Supplementary Table 1. Overview of the optimized hyperparameters for each clustering algorithm 5](#_Toc202350656)

[Supplementary Table 2. Performance of the clustering algorithms 5](#_Toc202350657)

[Supplementary Figure 2. Predictor factors of the best data-driven model (Spectral Clustering) 6](#_Toc202350658)

[Supplementary Table 3. Effect sizes of the clinical and socio-demographic characterization of the PD subgroups 7](#_Toc202350659)

The resulting z-score distributions for people with Parkinson’s disease (PwPD) and controls, obtained by the statistical analyses described in Methods section, showed that PwPD had greater negative deviations from the norm than controls, indicating greater cognitive impairment in the five cognitive domains investigated. The distribution of z-scores was mostly within the expected range, with few individuals exceeding the [3 to -3] interval, assuming a normal distribution in the group (see Supplementary Panel 1 below).

| **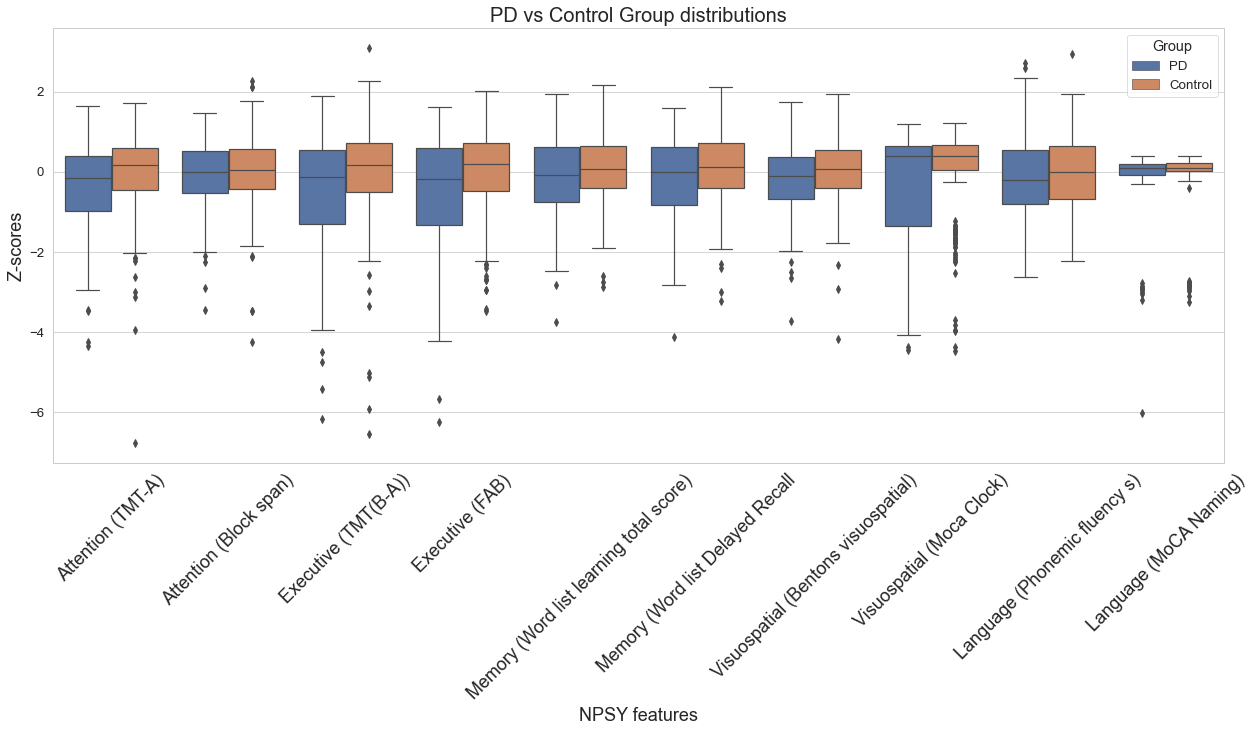** | | | |
| --- | --- | --- | --- |
| **Domains** | **Features** | **PD group (n=115) Mean (SD)** | **Control group (n=226) Mean (SD)** |
| **Attention** | TMT-A | -0.40 (1.14) | -0.02 (0.98) |
|  | Block Span | -0.10 (0.87) | -0.01 (0.92) |
| **Executive** | TMT (B-A) | -0.51 (1.55) | 0.01 (1.23) |
|  | FAB | -0.57 (1.51) | 0.005 (1) |
| **Memory** | Word list learning total score | -0.12 (1.01) | 0.06 (0.83) |
|  | Word list delayed recall | -0.20 (1.03) | 0.05 (0.89) |
| **Visuospatial** | Judge Line of Orientation | -0.21 (0.89) | -0.0016 (0.81) |
|  | MoCA clock | -0.23 (1.41) | 0.017 (1.11) |
| **Language** | Phonemic fluency S | -0.03 (1.03) | 0.026 (0.90) |
|  | MoCA Naming | -0.18 (0.96) | -0.043 (0.69) |

**Supplementary Panel 1. Z-score distribution of neuropsychological assessments within the PD and control group.** The figure and the table above provide an overview and a comparison of the z-score distribution (impairment profile) in the study population cohort. The study population cohort consists of 226 controls and 116 individuals with idiopathic Parkinsons disease (PD) from the Luxembourg Parkinsons Study. Abbreviations: Frontal Assessment Battery (FAB), Montreal Cognitive Assessment (MoCA), standard deviation (SD), Trail Making Test (TMT).


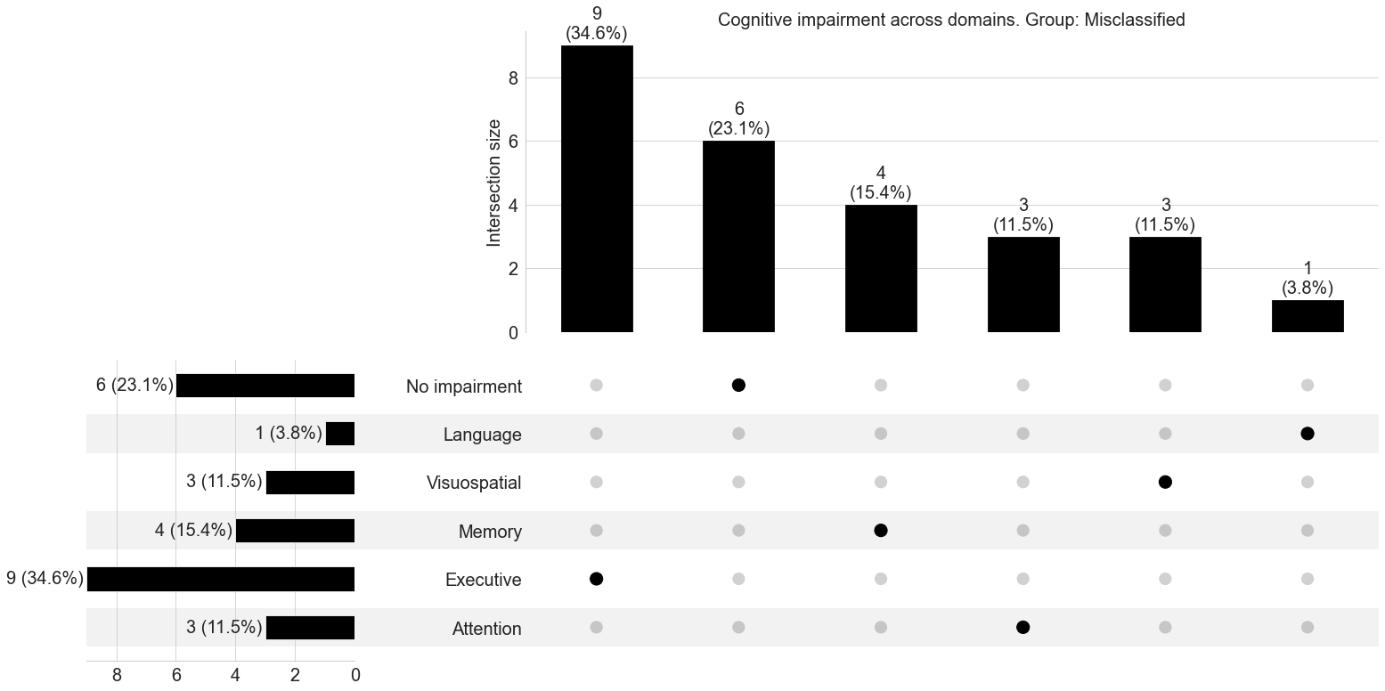

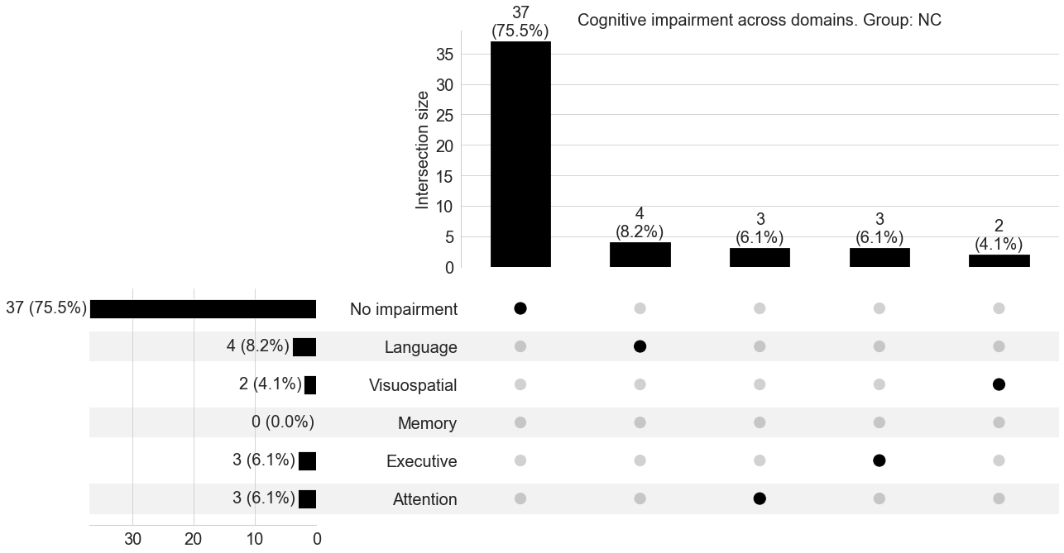


B)

A)


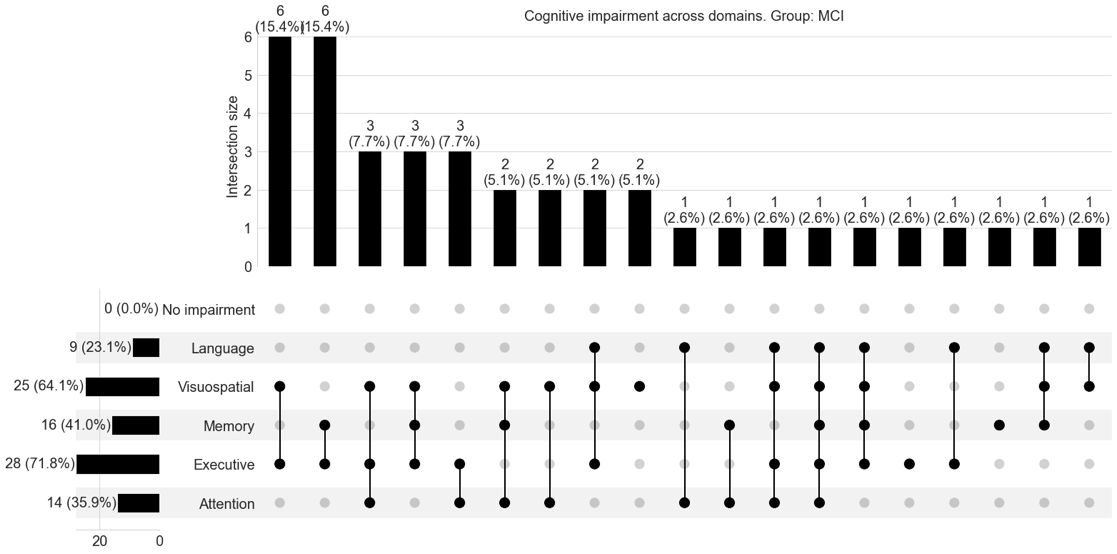


C)

**Supplementary Figure 1. Cognitive characterization and impairment profiles across the different PD subgroups.** A) Cognitive impairment profile of the group with normal cognition (NC) (n=49), participants with PD classified as NC by both diagnostic tools, B) cognitive impairment profile of the data-driven early-MCI group, participants with PD classified as having mild cognitive impairment (MCI) (n=26) by the data-driven model and as having a NC by the clinical diagnostic reference test (MDS PD-MCI level II), and C) cognitive impairment profile of the PD group with MCI, participants with PD classified as MCI by both diagnostic tools (n=39). This figure presents the number and percentage of individuals with PD with cognitive impairment within each subgroup across five cognitive domains: attention, executive function, visuospatial skills, memory, and language. An individual was classified as impaired in a domain if the z-score was at least 1.5 standard deviations below the age-adjusted normative values in any of the tests assessing that domain. A similar analysis was not conducted for the data-driven early-MCI group, consisting of one (1) participant with PD classified as NC by the data-driven model and as MCI by the clinical diagnostic reference test.

|  |  | Parameters | Range | Optimal value |
| --- | --- | --- | --- | --- |
| Models | SC | N clusters | [2,3,4] | 2 |
|  |  | Assign labels | [kmeans,discretize,cluster_qr] | kmeans |
|  |  | Affinity | [nearest_neighbors,rbf] | Nearest neighbors |
|  |  | Random state | 64 | 64 |
|  | GMM | N components | [2,3,4] | 2 |
|  |  | Covariance type | [full, tied, diag, spherical] | Diag |
|  |  | Init params | [kmeans,random] | Random |
|  |  | Max iterations | [100,1000,10000] | 100 |
|  |  | Reg covar | 1e-1 | 1e-1 |
|  |  | Random state | 64 | 64 |
|  | K-Means | N clusters | [2,3,4] | 2 |
|  |  | Max iterations | [100,1000,10000] | 100 |
|  |  | Algorithm | [elkan,auto,full] | Elkan |
|  |  | N init | [1,5,10,100] | 100 |
|  |  | Random state | 64 | 64 |

**Supplementary Table 1. Overview of the optimized hyperparameters for each clustering algorithm.** Abbreviations: Gaussian Mixture Model (GMM), Spectral Clustering (SC).

|  |  | Models | | |
| --- | --- | --- | --- | --- |
|  |  | SC* | K-Means | GMM |
| Metrics | Global accuracy | 0.76 | 0.79 | 0.80 |
|  | Sensitivity | 0.81 | 0.77 | 0.74 |
|  | MCI sensitivity | 0.97 | 0.65 | 0.55 |
|  | Precision | 0.79 | 0.79 | 0.79 |
|  | AUC | 0.81 | 0.74 | 0.77 |

**Supplementary Table 2. Performance of the clustering algorithms.** The table shows the performance metrics of the best models of each clustering algorithm in replicating the labels from the clinical diagnostic reference test (MDS PD-MCI level II criteria), widely considered as gold standard. Abbreviations: Area under the Receiver Operating Characteristic curve (AUC), Gaussian Mixture Model (GMM), Spectral Clustering (SC), best performing model (*).

**
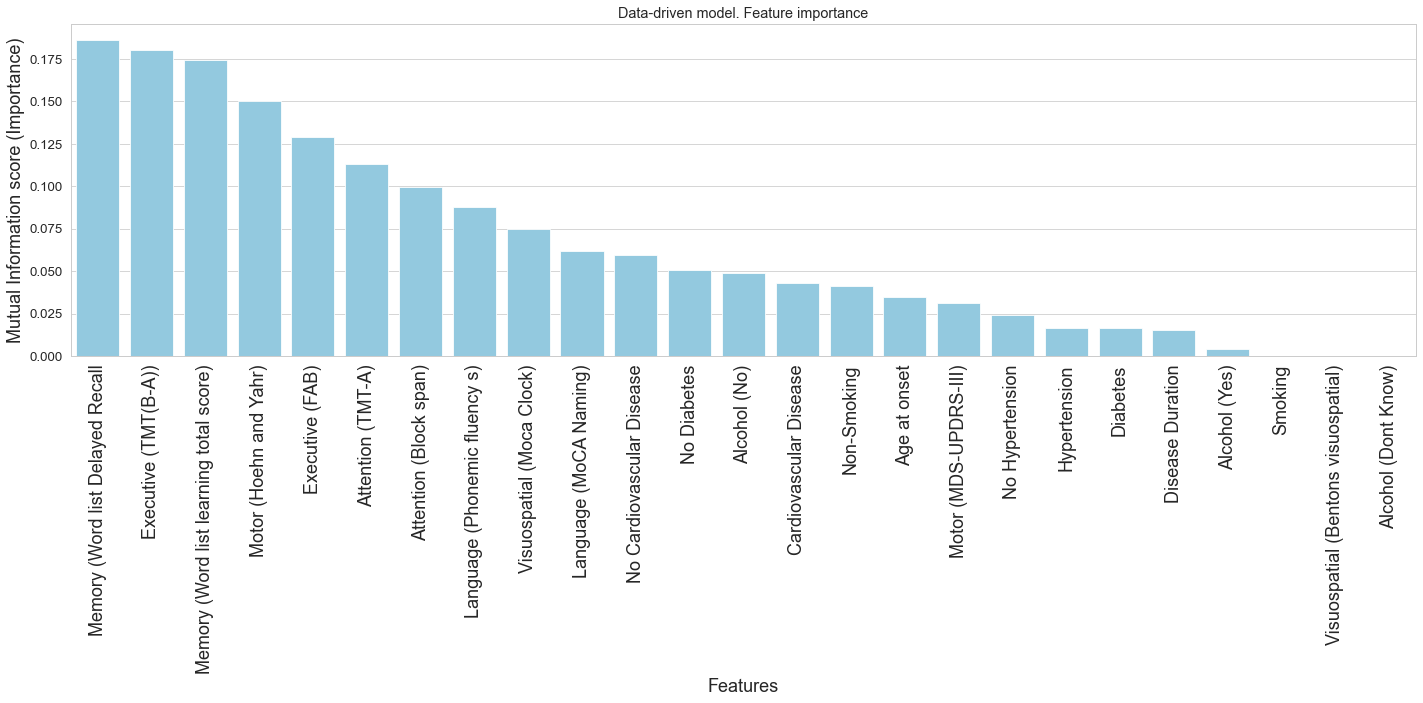
**

**Supplementary Figure 2. Predictor factors of the best data-driven model (Spectral Clustering).** This figure presents the relevance in the classification process of every feature introduced in the clustering algorithm**.** The clinical characteristics showed in the graph covered from domain-specific cognitive assessments (e.g., TMT B-A, FAB, …), disease-related factors (e.g., age at diagnosis, disease duration), comorbidities (e.g., cardiovascular disease, diabetes), and other clinical characteristics (e.g., MDS-UPDRS III, Hoehn and Yahr stage). Feature importance rankings derived from the best performing data-driven model (Spectral Clustering) using mutual information scores. Abbreviations: Trail Making Test Part (TMT), Frontal Assessment Battery (FAB), MDS-Unified Parkinsons Disease Rating Scale part III (MDS-UPDRS III), Montreal Cognitive Assessment (MoCA).

| **Features** | **NC vs**  **data-driven early-MCI** | **Data-driven early-MCI vs MCI** | **NC vs MCI** |
| --- | --- | --- | --- |
| **Socio-demographic** | | | |
| Age | 0.56 | 0.06 | 0.88 |
| Years of Education | -0.32 | -0.01 | -0.33 |
| **Clinical** | | | |
| **Cognitive Performance** | | | |
| TMT-A | -1.35 | -0.06 | -1.03 |
| Block span | -0.58 | -0.44 | -1.08 |
| TMT(B-A) | -1.39 | -0.47 | -1.91 |
| FAB | -1.09 | -0.67 | -1.56 |
| Word list learning total score | -1.64 | -0.19 | -1.99 |
| Word list Delayed Recall | -1.42 | -0.26 | -1.73 |
| Judgment of Line Orientation | -0.18 | -0.72 | -0.90 |
| MoCA clock | 0.80 | 1.16 | 2.06 |
| Phonemic fluency S | -0.09 | -0.32 | -0.39 |
| MoCA Naming | 0.03 | 0.61 | 0.78 |
| **Motor** | | | |
| Hoehn and Yahr | 1.18 | 0.66 | 0.81 |
| MDS-UPDRS-III | 0.43 | 0.18 | 0.60 |
| **ADL** | | | |
| PDQ-39 subitems 11-16 | 0.09 | -0.034 | 0.05 |
| **Neuropsychiatric symptoms** | | | |
| Depression (BDI-I) | 0.78 | -0.68 | 0.11 |
| Apathy (SAS) | 1.10 | -0.52 | 0.50 |
| **PROMs** | | | |
| Subjective Cognitive Complaints  (PDQ-39 subitems 30-33) | 1 | -0.26 | 0.75 |
| **Disease-related factors** | | | |
| Disease duration | 0.51 | -0.20 | 0.29 |
| Age at PD onset | 0.34 | 0.13 | 0.65 |

**Supplementary Table 3. Effect sizes of the clinical and socio-demographic characterization of the PD subgroups.** The table provides the quantitative differences between the three groups: normal cognition (NC), data-driven early-MCI and mild cognitive impairment (MCI) group. The magnitude of the observed differences was reported using effect sizes. For numerical variables that follow a normal distribution, Cohen's d was used. For numerical variables that do not follow a normal distribution, point biserial correlation was selected and then standardized into Cohen's d for ease of interpretation. Cramer’s V was used for ordinal features such as Hoehn and Yahr and MDS-UPDRS III. Abbreviations: activities of daily living (ADL), Becks Depression Inventory (BDI-I), Frontal Assessment Battery (FAB), MDS-Unified Parkinsons Disease Rating Scale part III (MDS-UPDRS III), Montreal Cognitive Assessment (MoCA), Parkinsons Disease Questionnaire (PDQ-39), Starkstein Apathy Scale (SAS), Trail Making Test Part (TMT).

**List of NCER-PD consortium members**:

Mariella GRAZIANO⁷, Alexandre BISDORFF⁵, Rene DONDELINGER⁵, Elodie THIRY³, Gelani ZELIMKHANOV³, Guy BERCHEM³, Liliana VILAS BOAS³, Linda HANSEN³, Martine GOERGEN³, Nancy DE BREMAEKER³, Nico DIEDERICH³, Romain NATI³, Roxane BATUTU³, Sylvia HERBRINK³, Jochen KLUCKEN¹,³, Rejko KRÜGER¹,²,³, Claire PAULY²,³, Lukas PAVELKA²,³, Marijus GIRAITIS²,³, Maria Fernanda NIÑO URIBE¹,³, Achilleas PEXARAS², Alexander HUNDT², Alexia MENDIBIDE², Ana Festas LOPES², Angelo FERRARI², Brian DEWITT², Carlos GAMIO², Estelle HENRY², Gaël HAMMOT², Geeta ACHARYA², Hermann THIEN², Ilsé RICHARD², Johanna TROUET², Kate SOKOLOWSKA², Katy BEAUMONT², Laura GEORGES², Lorieza CASTILLO², Lucie REMARK², Maeva MUNSCH², Margaux HENRY², Maud THERESINE², Olga KOFANOVA², Olivia ROLAND², Pauline LAMBERT², Saïda MTIMET², Wim AMMERLANN², Anne GRÜNEWALD¹, Armin RAUSCHENBERGER¹,², Clarissa P. C. GOMES¹, Dheeraj REDDY BOBBILI¹, Ekaterina SOBOLEVA¹,³, Elisa GÓMEZ DE LOPE¹, Enrico GLAAB¹, Evi WOLLSCHEID-LENGELING¹, Francoise MEISCH¹, Giuseppe ARENA¹, Ibrahim BOUSSAAD¹, Jens SCHWAMBORN¹, Kirsten ROOMP¹, Laure PAULY², ¹⁰, Michael T. HENEKA¹, Michele BASSIS¹, Muhammad ALI¹, Jade JABER¹,³, Patricia MARTINS CONDE¹, Patrick MAY¹, Paul WILMES¹, Piotr GAWRON¹, Rebecca TING JIIN LOO¹, Reinhard SCHNEIDER¹, Ruxandra SOARE¹, Sabine SCHMITZ¹, Sarah NICKELS¹, Sascha HERZINGER¹, Sinthuja PACHCHEK¹, Soumyabrata GHOSH¹, Stefano SAPIENZA¹, Valentin GROUES¹, Venkata SATAGOPAM¹, Iñigo YOLDI BERGUA¹, Gabriel MARTINEZ TIRADO¹, Jochen OHNMACHT², Anne-Marie HANFF², ¹⁰, ¹¹, Carlos VEGA², Chouaib MEDIOUNI², Deborah MCINTYRE², Eduardo ROSALES², Fozia NOOR², Gessica CONTESOTTO², Gloria AGUAYO², Guilherme MARQUES², Jérôme GRAAS², Joëlle FRITZ², Magali PERQUIN², Manon GANTENBEIN², Maura MINELLI², Michel VAILLANT², Myriam ALEXANDRE², Myriam MENSTER², Olena TSURKALENKO², Raquel SEVERINO², Sibylle BÉCHET³, Tainá M. MARQUES², Ulf NEHRBASS², Victoria LORENTZ², Zied LANDOULSI², Sonja JÓNSDÓTTIR², David BOUVIER⁴, Katrin FRAUENKNECHT⁴, Michel MITTELBRONN¹, ², ⁴, ¹⁰, ¹², ¹³, Roseline LENTZ⁶, Jean-Paul NICOLAY⁹, Nadine JACOBY⁸

1 Luxembourg Centre for Systems Biomedicine, University of Luxembourg, Esch-sur-Alzette, Luxembourg

2 Luxembourg Institute of Health, Strassen, Luxembourg

3 Centre Hospitalier de Luxembourg, Strassen, Luxembourg

4 Laboratoire National de Santé, Dudelange, Luxembourg

5 Centre Hospitalier Emile Mayrisch, Esch-sur-Alzette, Luxembourg

6 Parkinson Luxembourg Association, Leudelange, Luxembourg

7 Association of Physiotherapists in Parkinsons Disease Europe, Esch-sur-Alzette, Luxembourg

8 Private practice, Ettelbruck, Luxembourg

9 Private practice, Luxembourg, Luxembourg

10 Faculty of Science, Technology and Medicine, University of Luxembourg, Esch-sur-Alzette, Luxembourg

11 Department of Epidemiology, CAPHRI School for Public Health and Primary Care, Maastricht University Medical Centre+, Maastricht, the Netherlands

12 Luxembourg Center of Neuropathology, Dudelange, Luxembourg

13 Department of Life Sciences and Medicine, University of Luxembourg, Esch-sur-Alzette, Luxembourg
